# Mobile element insertions in rare diseases: a comparative benchmark and reanalysis of 60,000 exome samples

**DOI:** 10.1038/s41431-023-01478-7

**Published:** 2023-10-19

**Authors:** Robin Wijngaard, German Demidov, Luke O’Gorman, Jordi Corominas-Galbany, Burcu Yaldiz, Wouter Steyaert, Elke de Boer, Lisenka E. L. M. Vissers, Erik-Jan Kamsteeg, Rolph Pfundt, Hilde Swinkels, Amber den Ouden, Iris B. A. W. te Paske, Richarda M. de Voer, Laurence Faivre, Anne-Sophie Denommé-Pichon, Yannis Duffourd, Antonio Vitobello, Martin Chevarin, Volker Straub, Ana Töpf, Anneke J. van der Kooi, Francesca Magrinelli, Clarissa Rocca, Michael G. Hanna, Jana Vandrovcova, Solve-RD consortium, Stephan Ossowski, Steven Laurie, Christian Gilissen

**Affiliations:** 1 Department of Human Genetics, Radboud University Medical Center, Nijmegen, The Netherlands; 2 Donders Institute for Brain, Cognition and Behaviour, Radboud University, Nijmegen, The Netherlands; 3 Universitätsklinikum Tübingen – Institut für Medizinische Genetik und angewandte Genomik, Tübingen, Germany; 4 Radboud Institute for Molecular Life Sciences, Radboud University Medical Center, Nijmegen, The Netherlands; 5 Centre de Référence Maladies Rares “Anomalies du développement et syndromes malformatifs”, Centre de Génétique, FHU-TRANSLAD et Institut GIMI, CHU Dijon Bourgogne, Dijon, France; 6 UMR1231-Inserm, Génétique des Anomalies du développement, Université de Bourgogne Franche-Comté, Dijon, France; 7 Laboratoire de Génétique chromosomique et moléculaire, UF6254 Innovation en diagnostic génomique des maladies rares, Centre Hospitalier Universitaire de Dijon, Dijon, France; 8 John Walton Muscular Dystrophy Research Centre, Translational and Clinical Research Institute, Newcastle University and Newcastle Hospitals NHS Foundation Trust, Newcastle upon Tyne, UK; 9 Department of Neurology, Amsterdam UMC, University of Amsterdam, Amsterdam Neuroscience, Amsterdam, The Netherlands; 10 Department of Clinical and Movement Neurosciences, UCL Queen Square Institute of Neurology, London, UK; 11 Department of Neuromuscular Diseases, UCL Queen Square Institute of Neurology, London, UK; 12 Clinical Pharmacology, William Harvey Research Institute, School of Medicine and Dentistry, Queen Mary University of London, London, UK; 13 Centro Nacional de Análisis Genómico (CNAG), Centre for Genomic Regulation (CRG), Barcelona Institute of Science and Technology (BIST), Barcelona, Spain

**Keywords:** Genetics, Computational biology and bioinformatics

## Abstract

Mobile element insertions (MEIs) are a known cause of genetic disease but have been underexplored due to technical limitations of genetic testing methods. Various bioinformatic tools have been developed to identify MEIs in Next Generation Sequencing data. However, most tools have been developed specifically for genome sequencing (GS) data rather than exome sequencing (ES) data, which remains more widely used for routine diagnostic testing. In this study, we benchmarked six MEI detection tools (ERVcaller, MELT, Mobster, SCRAMble, TEMP2 and xTea) on ES data and on GS data from publicly available genomic samples (HG002, NA12878). For all the tools we evaluated sensitivity and precision of different filtering strategies. Results show that there were substantial differences in tool performance between ES and GS data. MELT performed best with ES data and its combination with SCRAMble increased substantially the detection rate of MEIs. By applying both tools to 10,890 ES samples from Solve-RD and 52,624 samples from Radboudumc we were able to diagnose 10 patients who had remained undiagnosed by conventional ES analysis until now. Our study shows that MELT and SCRAMble can be used reliably to identify clinically relevant MEIs in ES data. This may lead to an additional diagnosis for 1 in 3000 to 4000 patients in routine clinical ES.

## Introduction

Mobile elements or transposable elements are interspersed repetitive genetic sequences found throughout eukaryotic genomes and characterized by their distinctive capacity to move to a new genomic location [1, 2]. Mobile elements can be separated into two main classes, transposons and retrotransposons, which differ in their transposition mechanism and among which only a fraction of the latter are active. Only retrotransposons are thus capable of creating new insertions, known as mobile element insertions (MEIs) [2–6].

The human retrotransposon class encompasses L1, Alu and SVA (SINE/VNTR/Alu) elements. All three elements present a poly-A tail at the end of their sequence and move through the same L1-dependent target-primed reverse transcription mechanism [2, 6], whereby retrotransposition of all three elements highly depends on the existence of functional and active L1 sequences [6]. Collectively, L1, Alu and SVA sequences account for almost 30% of the human genome, with approximately 500,000 L1 sequences, 1,100,000 Alu sequences and 3000 SVA sequences identified [5, 6]. The vast majority of L1 sequences have lost their retrotransposability due to accumulated genetic variation in their sequence, leaving only 80 to 100 active L1 sequences in the human genome [7–9].

Active mobile elements act as insertional mutagens and can lead to genetic diseases when inserted at points in the genome that disrupt gene function [2–4]. Based on the frequency of identified disease-causing de novo MEIs and genomic comparisons between individuals, studies have repeatedly estimated that novel MEI events in the genome occur in 1/20 live births, with a higher frequency for Alu than L1 and SVA [4, 6, 10–12]. More than 120 MEI events have so far been associated with human disease [2]. Studies have shown that the overall frequency of disease-causing MEI findings in ES data, as demonstrated by resequencing analysis of large datasets, is consequently rather low, being approximately between 1 in 2500 to 3000 [13–15]. Nevertheless, identification of MEIs may yield new diagnoses, especially when working with large cohorts.

Identification of MEIs in sequencing data relies on the use of discordant pairs (DP) and/or clustered split reads or clipped reads (SR), mostly in combination, and a library of consensus sequences of known archaic MEIs in the human reference genome. A large number of tools is currently available, but most of these have been developed specifically for the analysis of genome sequencing (GS) data. Due to the targeted nature of exome sequencing (ES), assumptions on the presence of SR and DP as made for GS may be violated and therefore also methods specifically for ES have also been developed [13, 14, 16, 17]. Although several benchmark studies are available for MEI detection in GS data, no such benchmark exists for ES [18, 19].

The goal of the Solve-RD project is to diagnose patients in whom inherited diseases are suspected but whose actual genetic diagnosis has remained unsolved, despite prior ES analysis having been undertaken. This is being realised through a comprehensive re-analysis of the existing ES data as well as the generation of new -omics data [20]. The reanalysis offers the possibility to mine ES data for additional genetic variants, such as MEIs, which are not routinely explored and detected, but likely explain a small fraction of unsolved cases. The objectives of this study were twofold: first, to evaluate and compare existing MEI detection tools on ES data, and second to apply to best tool(s) to 10,890 samples from the Solve-RD reanalysis cohort in addition to 52,624 samples from Radboudumc in order to identify the genetic cause of disease in unsolved patients.

## Materials and methods

### MEI detection tools

We selected tools developed in recent years that could potentially provide reliable results from ES data. All tools had been developed or previously tested on human data and their intended use was to search for non-reference MEIs (i.e. MEIs not present in the reference genome) in individual samples. This led to the inclusion of a total of six tools: ERVcaller [21], MELT [14, 16], Mobster [17], SCRAMble [13], TEMP2 [22], and xTea [23] (Table 1). SCRAMble and Mobster were specifically designed for MEI detection in ES data, while MELT was designed for GS but additionally validated for ES. ERVcaller and xTea state in their respective documentation that ES data is a possible input type, while TEMP2 was the only GS-specific tool.

**Table 1:** Overview of features of the mobile element detection tools evaluated in this study.

Tool	Type of reads^a^	Input	Output	ES^b^	Year, Reference	Source
ERVcaller	Discordant pairs + Split or clipped reads	FASTQ or BAM	VCF file	Yes	2019, ^21^	https://github.com/xunchen85/ERVcaller
MELT	Discordant pairs + Split or clipped reads	BAM (aligned with BWA) or CRAM	VCF file	Yes	2016, ^14,16^	https://melt.igs.umaryland.edu
Mobster	Discordant pairs + Split or clipped reads	BAM	Text file	Yes	2014, ^17^	https://github.com/jyhehir/mobster
SCRAMble	Split or clipped reads	BAM	VCF file	Yes	2020, ^13^	https://github.com/GeneDx/SCRAMble
TEMP2	Discordant pairs + Split or clipped reads	FASTQ or BAM	BED file	No	2021, ^22^	https://github.com/weng-lab/TEMP2
xTea	Discordant pairs + Split or clipped reads	BAM or CRAM	VCF file	Yes	2021, ^23^	https://github.com/parklab/xTea

^a^Type of reads used by the algorithm to detect MEIs in sequencing data.

^b^The ES column specifies whether the tool can be used for ES data as described by the author.

Abbreviations: *ES*, exome sequencing; *MEI*, mobile element insertion.

### Exome benchmarking datasets

We assessed the tools for the detection of MEIs located within or in close proximity to exons, defined as events occurring within 50 bp of the exon boundaries (hereafter referred to as target regions). Two distinct and independent datasets were used for this purpose. A detailed description of the creation of both datasets can be found in the Supplementary Materials and Methods.

Exome dataset 1 consisted of 20 exome samples wherein reference MEIs were curated using PacBio HiFi long-read sequencing of the same samples using PALMER [24–26]. This final dataset contained a total of 256 reference MEI calls in target regions. Among these calls, 242 were Alu, 11 L1 and 3 SVA (Supplementary Table 1).

Exome dataset 2 was obtained through manual curation of high-confidence MEI calls in 100 trio exome samples. This exome benchmark dataset was created by merging calls derived from the six distinct tools, followed by rigorous filtering and visual inspection in the Integrative Genomics Viewer (IGV) [27]. The dataset contained 1111 reference MEI calls, of which 907 were Alu, 182 L1 and 22 SVA (Supplementary Table 2).

### Genome benchmarking datasets

For comparison, the tools were also evaluated with GS data using two well-characterised human genome samples: HG002 and NA12878. The HG002 sample has recently been comprehensively characterised using multiple sequencing technologies, including long-read sequencing, and is currently the best available reference sample for structural variants benchmarking [28]. Reference MEI calls, generated using PALMER as a component of the NIST Genome In a Bottle high-confidence structural variants dataset, were extracted from the study of Torene et al. [13]. The sample contained 1467 MEIs, of which 1237 were Alu, 157 were L1 and 73 were SVA. Structural variants in the NA12878 sample, including MEIs, have been characterized and published as part of the integrated structural variant map of the 1000 Genomes Project phase 3 data [29]. By selecting MEI calls, we obtained a reference set of 1092 MEI calls, out of which 922 were Alu, 124 were L1 and 46 were SVA.

Data from both samples were downloaded in FASTQ format from the Genome in a Bottle consortium (https://github.com/genome-in-a-bottle/giab_data_indexes) and consisted of 150 bp paired-end sequencing. Reads from two flow cells (run 1 and run 2) were aligned with the Burrow-Wheeler-Aligner (BWA) v.07.13 using the GRCh37 as reference assembly to achieve ~40X coverage according to the GIAB data description.

### Tool usage and optimization

All tools were run with default parameters. MELT was run in single mode and using the “–exome” flag on the exome samples. For TEMP2, the midpoint between start and end position was used as breakpoint.

In order to improve the performance of the tools and decrease the false positive (FP) rate, we applied two different filtering strategies to the output of each tool:

- Common strategy: in this case, a uniform filter was used based on the total number of reads supporting the MEI (SR + DP) and the number of SRs. We defined a threshold of at least five supporting reads with two SRs for exome samples, and ten supporting reads with two SRs for genome samples.
- Optimised strategy: in this approach, optimal filtering thresholds were determined using exome dataset 1 and the HG002 genome sample. A subset of parameters, focusing on either the quantity of supporting reads for the MEI or the quality of the call, was chosen (Supplementary Table 3). Multiple combinations of these parameters were tested to identify the most suitable configuration, determined by the highest achieved performance (F-score). Subsequently, these parameters were applied on exome dataset 2 and the NA12878 sample.

### Statistical analysis

MEI predictions from each tool were compared against the reference set. For ES samples, only MEIs within target regions (a 50 bp window around exon boundaries) were included in the analysis. True positives (TP) were defined as MEI calls located within a window of +/− 10 bp around the TSD of a reference insertion site. In cases where the TSD of the reference call was unknown, a range of +/− 50 bp from the reference insertion site was allowed. Any predicted MEIs outside of these regions were considered FP calls. False negative (FN) calls were defined as the absence of any predicted MEI within the defined range of a reference insertion site.

Tools were evaluated on their precision, sensitivity, and F-score. Sensitivity was calculated as TP/(TP + FN), precision as TP/(TP + FP) and F-score as 2x[(precision x sensitivity) / (precision + sensitivity)].

### Solve-RD cohort and Radboudumc cohort

The Solve-RD cohort included exome samples from 10,890 individuals, including 6247 affected cases from 6231 unrelated families. The remainder of the samples were unaffected relatives and samples submitted to the Solve-RD cohort for validation purposes. Samples were collected from multiple centres across Europe which included 1835 (29.4%) ERN-ITHACA (Intellectual disability, TeleHealth And Congenital Anomalies), 2605 (41.7%) ERN-RND (Rare Neurological Diseases), 1457 (23.3%) EURO-NMD (ERN for NeuroMuscular Diseases) and 350 (5.6%) ERN-GENTURIS (GENetic TUmour RIsk Syndromes) index cases. Human phenotype ontology (HPO) data and ES data for all patients were obtained. All ES data submitted was analysed in an identical fashion, to avoid any batch effects, using the RD-Connect Genome-Phenome Analysis Platform (GPAP) standard analysis pipeline [30]. Exome capture was performed using 28 different kits. FASTQs were aligned using BWA-MEM v0.7.8 to the decoy version of GRCh37 (hs37d5) as used by the 1000 Genomes project.

The Radboudumc dataset consisted of 52,624 exome samples from 35,488 affected cases across 33,509 unrelated families. Among these, 8861 were complete parent-child trios. Patients were referred for genetic testing for any clinical indication requiring exome analysis, e.g. intellectual disability, hereditary cancer syndromes, movement disorders or blindness. Samples were processed and analysed as previously described in Lelieveld et al. [31]. Briefly, DNA was isolated from whole blood and exome capture was performed using Agilent SureSelect v4 (*n* = 5588), Agilent SureSelect v5 (*n* = 37,803) and Twist v1 (*n* = 9233). Samples were sequenced with 2 × 150 bp reads on an Illumina HiSeq 2000/4000 instrument or NovaSeq 6000 instrument. Sequence reads were aligned to the GRCh37 reference genome using BWA version v0.7.12 and duplicate marked using Picard v1.90.

Patient samples, together with a basic phenotype description and molecular diagnosis (when available), were analysed in an anonymous fashion.

### Identification of disease-causing MEIs

In order to identify MEIs relevant to disease in the Solve-RD and Radboudumc cohort, we applied the following filtering strategy: first, MEI were limited to those that fell within a window of +/− 50 bp of ES target areas. All MEIs in non-affected cases were removed, followed by the exclusion of MEIs present in the retrotransposon insertion polymorphisms in humans (dbRIP) database [32]. MEI frequency was calculated by counting all overlapping (+/− 50 bp) MEIs in the cohort and only rare events, defined as having a frequency <0.03% in their respective cohorts, were retained. We further filtered by only considering MEIs found in clinically relevant genes based on the patient’s phenotype as defined by the original requested gene panel or ERN group. For the Radboudumc cohort, in addition, only MEIs at exonic and splice sites were selected. The remaining MEIs were visually inspected in IGV to discard low-quality calls. Finally, MEIs were selected for confirmation by considering the phenotype-genotype match, inheritance pattern and presence of a second variant in the case of an autosomal recessive disorder.

### MEI validation and diagnoses

All potential diagnostic MEIs were validated by complementary laboratory methods. MEIs found in the Solve-RD cohort were validated at the centre of their respective submitter. Samples from Radboudumc were validated in-house. A detailed description can be found in the Supplementary Materials and Methods. Validated MEIs were evaluated by a clinical laboratory specialist and a physician, and a certified diagnostic report was issued for all cases diagnosed in this study.

## Results

### Exome benchmark

**Fig. 1:**
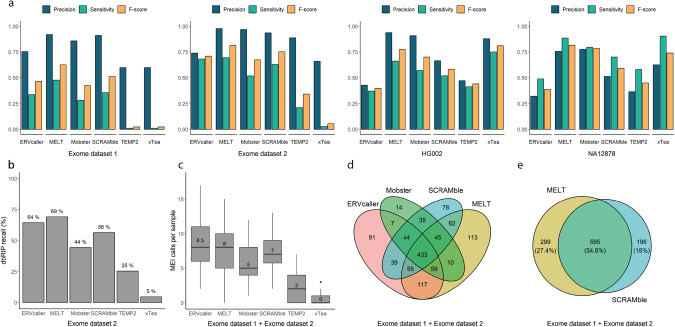
Overview of MEI benchmark results. **a** Comparison of tool performance for detecting MEIs in the exome and genome datasets, showing the achieved precision, sensitivity and F-score. The best results achieved among the different filtering strategies are represented (Supplementary Tables 3 and 5). **b** Boxplots of the distribution of mobile element insertion (MEI) calls per sample in the exome datasets, located within target regions. To improve visibility, two data point from ERVcaller (with call counts of 39 and 100) have been omitted from the plot. **c** Recall of MEIs described in the dbRIP database (known human MEI polymorphisms) included in the reference set of exome dataset 2. **d** Overlap of true positive calls between ERVcaller, MELT, Mobster and SCRAMble across both exome datasets. **e** Overlap of true positive calls between MELT and SCRAMble across both exome benchmark datasets.

We generated and evaluated the tools on two independent ES benchmark datasets (Fig. 1a). Optimised parameter selection and results per filtering strategy for both datasets are summarised in Supplementary Table 4 and Supplementary Table 5. Mean runtimes for each tool are depicted in Supplementary Fig. 1.

When considering the most favorable parameters achieved across strategies, MELT yielded the best overall performance on both datasets (Supplementary Table 5). Its precision ranged between 0.92 and 0.98, and its sensitivity between ranged between 0.48 and 0.69. Followed by SCRAMble, ERVcaller and Mobster, which showed similar precision values between 0.74 and 0.97 and sensitivity values between 0.28 and 0.68. In contrast, xTea and TEMP2 performed poorly on ES data with sensitivities below 0.2. The latter two algorithms detected on average between zero and two MEI calls per sample which was also considerably lower than the other methods (Fig. 1c). MELT, ERVcaller and SCRAMble also exhibited the highest sensitivities for the detection of previously reported MEIs in the dbRIP database, included in exome dataset 2. Specifically, MELT achieved a recall of 69%, ERVcaller of 64% and SCRAMble of 56% (Fig. 1b).

Among the four most reliable tools (ERVcaller, MELT, Mobster, and SCRAMble), distinct reference MEIs were successfully identified in both exome datasets. The limited concordance of their results emphasizes the possibility of improving MEI detection rates through the simultaneous use of several tools (Fig. 1d). The combination of MELT and SCRAMble achieved the highest detection rate of 1090 out of the 1367 reference calls (79.7%). It is noteworthy that there was only a 54.6% concordance between the tools (Fig. 1e, Supplementary Table 6).

### Genome benchmark

We also compared the performance of these methods on two well-characterised genome samples (HG002 and NA12878) with different filtering strategies (Supplementary Table 4 and Supplementary Table 7).

We found that the results obtained in both samples were very comparable. However, almost all tools achieved a slightly higher sensitivity but lower precision on the NA12878 sample (Fig. 1a). MELT and xTea outperformed the other methods with an average precision of 0.75 and 0.84 and an average sensitivity of 0.77 and 0.83, respectively. MELT performed very consistently in ES and GS data. In contrast, xTea yielded striking differences in performance between ES and GS data, with a high number of FNs in ES data due to the overall low detection rate of MEIs, resulting in a substantially lower sensitivity.

Mobster and SCRAMble achieved intermediate results, with sensitivities and accuracies between 0.6 and 0.8. TEMP2 and ERVcaller performed worse than the other tools, mainly because these tools were more imprecise in determining the exact insertion point, which made them fall out of the range established for considering the MEI call to be correct (Supplementary Fig. 2). This led to low precisions of between 0.3 and 0.5 in GS data, combined with low sensitivities of between 0.4 and 0.5.

### Solve-RD and Radboudumc cohorts

**Fig. 2:**
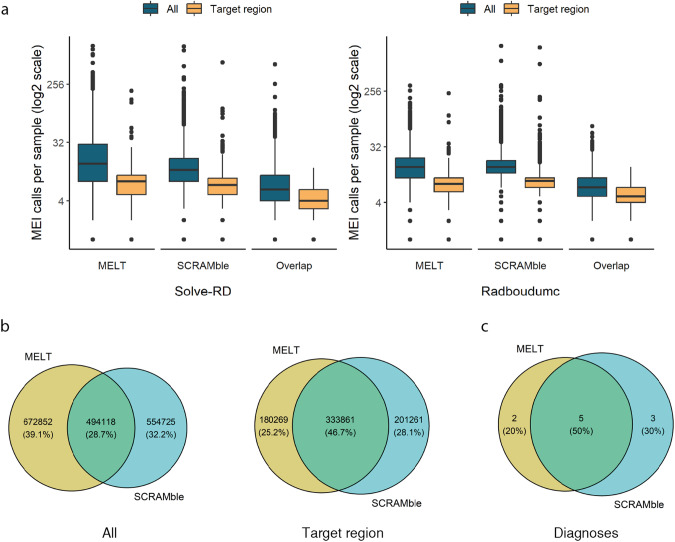
Results from the reanalysis of the Solve-RD and Radboudumc cohorts. **a** Boxplots showing the number of calls identified per sample, both across all calls and those limited to target regions (within 50 bp range around exon boundaries), within the Solve-RD and Radboudumc cohorts. Note that the X scale is log2. **b** Overlap of MEI events detected in the Solve-RD and Radboudumc cohorts by MELT and SCRAMble, including both all calls and those restricted to target regions. **c** Overlap of disease-causing MEIs described in the study between MELT and SCRAMble.

Based on the ES benchmarking, we selected both MELT and SCRAMble to search for new possible disease-causing MEIs in the Solve-RD and Radboudumc cohorts. Calls with less than five supporting reads and two SRs were filtered out, and for MELT, also calls with the “ac0” flag. By merging individual tool sets with a +/− 50 bp tolerance, a total of 389,575 MEI calls were detected in the Solve-RD cohort (MELT: 299,341; SCRAMble: 174,126; overlap: 83,892) and 1,332,120 in the Radboudumc cohort (MELT: 867,629; SCRAMble: 874,717; overlap: 410,226) (Fig. 2b). Across both cohorts, 25,080 unique MEI sites (Alu: 16,097; L1: 7,443; SVA: 1,540) were identified. The median number of calls per individual, after combining MELT and SCRAMble, was 23 (interquartile range: 17–30) of which 11 (interquartile range: 9–13) fell within target regions (Fig. 2a).

Using our filtering approach, 296 MEIs from the Solve-RD cohort and 432 exonic and splicing MEIs from the Radboudumc cohort were manually inspected in IGV and correlated with patient’s phenotype (Supplementary Table 8). A total of 15 potential candidates were further explored and validated in the laboratory by orthogonal methods resulting in 10 unique disease-causing MEIs (Table 2). The remaining five were excluded as either being considered benign (*n* = 3) or not confirmed in the laboratory (*n* = 2). Diagnosis rates between cohorts were not statistically different (Fisher Exact test *p* > 0.05), with three confirmed diagnoses in the Solve-RD cohort (3/6247 = 0.048% CI 95% [0.000%–0.102%]) and seven in the Radboudumc cohort (7/33,509 = 0.021% CI 95% [0.005%–0.036%]).

**Table 2:** Disease causing MEIs identified in the Solve-RD and Radboudumc cohorts.

Gene	gHGVS	cHGVS	Intron/Exon	MEI type	Strand	Cohort	Tool
*APC*	chr5(GRCh37):g.112173754_112173755insL1	NM_000038 c.2463_2464insL1	Exon 16/16	L1	reverse	Radboudumc	SCRAMble/MELT
*AVPR2*	chrX(GRCh37):g.153171115_153171116insAlu	NM_000054 c.155_156insL1	Exon 2/3	L1	reverse	Radboudumc	SCRAMble/MELT
*BRCA2*	chr13(GRCh37):g.32969020_32969020insAlu	NM_000059 c.9451_9452insAlu	Exon 25/28	Alu	reverse	Radboudumc	MELT
*CC2D2A*	chr4(GRCh37):g.15517501_15517502insAlu	NM_001080522 c.981_982insAlu	Exon 11/38	Alu	forward	Radboudumc	MELT
*COL11A1*	chr1(GRCh37):g.103471853_103472303del	NM_001854 c.1684-431_1702del	Exon 16/67	na	na	Radboudumc	SCRAMble
*COL6A2*	chr21(GRCh37):g.47536717_47536718insAlu	NM_001849 c.988_989insAlu	Exon 10/28	Alu	forward	Solve-RD	SCRAMble/MELT
*NIBPL*	chr5(GRCh37):g.37020844_37020845insAlu	NM_133433 c.5226-33InsAlu	Intron 26 (47 exons)	Alu	reverse	Solve-RD	SCRAMble/MELT
*NKX2-1*	chr14(GRCh37):g.36987132_36987133insAlu	NM_001079668 c.556_557InsAlu	Exon 3/3	Alu	reverse	Solve-RD	SCRAMble
*TTN*	chr2(GRCh37):g.179593380_179593381insAlu	NM_133378 c.15540_15541insAlu	Exon 63/312	Alu	reverse	Radboudumc	SCRAMble/MELT
*USH2A*	chr1(GRCh37):g.216019288_216019289insAlu	NM_206933 c.8933_8934insAlu	Exon 45/72	Alu	reverse	Radboudumc	SCRAMble

*MEI* mobile element insertion, *na* not applicable.

**Fig. 3:**
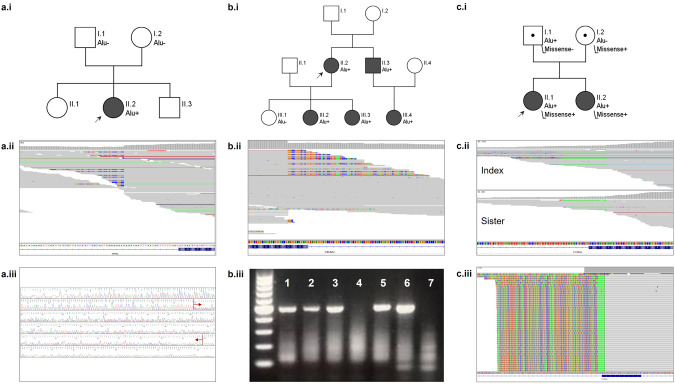
Three examples of disease-causing mobile element insertions (MEIs) found in our study. **a.i** Family pedigree of *NIPBL* case from the Solve-RD cohort, showing that the MEI was found de novo in the index patient, with both parents testing negative for the insertion. **a.ii** Screen capture from the integrative genomics viewer (IGV) showing the MEI as seen in ES data. Note the presence of split reads (SR), discordant pairs (DP) and the poly-A tail, which is viewed as poly-T as the Alu element is inserted on the reverse strand. **a.iii** Results of the MEI confirmation by PCR and Sanger sequencing. The red arrows delimit the Alu sequence. **b.i** Family pedigree of *COL6A2* case from the Solve-RD cohort, showing the expected co-segregation of the MEI with disease phenotype in the family, in an autosomal dominant manner. **b.ii** Screen capture from IGV of the MEI as seen in ES data. **b.iii** Results of the MEI confirmation by primer specific-PCR. These primers produce an amplicon when the Alu sequence is present (lanes 1–3 and 5–6) but not when is absent (lanes 4 and 7 (control DNA)). **c.i** Family pedigree of *CC2D2A* case from the Radboudumc cohort. Both siblings are affected with the same phenotype showing a typical molar tooth sign on MRI. The disease is inherited in an autosomal recessive manner with the father being a carrier of the Alu variant, and the mother the carrier of a pathogenic single nucleotide variant. **c.ii** Screen capture from IGV of the MEI as seen in ES data. Note that only the poly-A sequence is captured by ES data, which is why only MELT was able to detect the insertion in the index case. The MEI in the sister of the index was a FN result for both tools. This was probably due to the lower sequencing depth (38× vs 23× at the breakpoint of the MEI) and the lower number of reads spanning the insertion (8 reads vs 4 reads). **c.iii** Results of the MEI confirmation by targeted long-read PCR and amplicon sequencing, showing the complete inserted Alu sequence.

The complete MEI sequence obtained by confirmatory analysis was compared with the consensus sequences of the Dfam database to confirm their homology [33]. Seven insertions were Alu elements and two were L1 elements. The MEIs were located in the following genes: *APC, AVPR2, BRCA2, CC2D2A, COL6A2, NIBPL, NKX2-1, TTN* and *USH2A*. In the *COL11A1* gene, instead of a MEI, a deletion near an ancient MEI site of 450 bp was found. The location of the deletion caused the SR to contain the sequence of an Alu element, which was subsequently identified as a MEI by the tools (Supplementary Fig. 3). When possible, the inheritance was determined and in two cases the MEI was a de novo event in the patient (*NIPBL* and *NKX2-1* cases). Three examples are shown in Fig. 3 and additional clinical information on the cases can be found in the Supplementary Results and Supplementary Table 9. The *NKX2-1* case has been described elsewhere [34].

As for the clinical sensitivity of both tools, MELT detected seven (70.0%) while SCRAMble detected eight (80.0%) of the 10 diagnostic cases (Fig. 2c). The concordance of both tools was of only 50.0% in this cohort. Therefore, their combination increased the diagnostic yield, as suggested based on the benchmark results. In addition, a FN was also detected for both tools, as a sibling of one patient carried the same MEI but neither tool was able to detect it from the ES data (Fig. 3c.ii). This MEI was also absent in the unfiltered tool data. The failure of the tools to detect this event is probably attributable to the lower mean sequencing depth which resulted in a lower number of reads covering the MEI.

## Discussion

Mobile elements have been generally understudied in patients with rare diseases due to difficulties in their detection and the technical limitations of genetic testing methods. However, MEIs can now be detected in ES data with relative ease thanks to bioinformatics tools designed for this purpose. Although numerous tools have been developed in recent years, only a few have been specifically designed for ES. In the present study, we have shown that there are significant differences in the performance of the tools between ES data and GS data, and that not all tools made for GS are suitable for ES despite their description specifying otherwise.

ES data presented some additional challenges compared to GS data that could affect the performance of the tools and may explain the observed differences. Firstly, ES data provides coverage primarily in targeted coding regions which causes MEIs to often be only partially captured. In contrast, in GS data, usually both the start and end of the MEI sequence are completely covered, which allows for a more comprehensive detection of typical MEI features such as TSD and poly-A stretches in many insertions. Such features provide more supportive evidence for the MEI event, thereby allowing more robust detection in GS compared to ES. Secondly, MEI detection calls rely on the identification and integration of SR and DP signatures. Although the number of SRs formed around the insertion might be similar in ES and GS data, the lack of coverage in non-coding regions leads to a lower number of DPs. These factors could hamper MEI detection as all tools except SCRAMble require at least one or two DPs to support the MEI in order to be identified.

According to our benchmark results, MELT was the most suitable tool for detecting MEIs in ES data, followed by SCRAMble, ERVcaller and Mobster. xTea and TEMP2 showed suboptimal performance on the ES data. In the case of xTea, it is possible that more intricate parameter tuning could improve its performance. On the other hand, TEMP2 was not designed for ES use, which could explain the observed results. In addition, with TEMP2 we observed that the reported MEI intervals were remarkably wide. This aspect could have influenced the results observed for the GS data, since most of the insertions tended to be near the extremes of the specified range and we used the midpoint for evaluation purposes (see Supplementary Fig. 2 for more information).

In general, filtering the obtained default calls improved the performance of all tools. A filtering strategy of a minimum of five (for ES) or ten (for GS) supporting reads with at least two SR seemed a valid strategy to improve precision without a substantial loss in sensitivity. Except for xTea, for which it had little effect as this algorithm already automatically adapts the threshold for the number of reads depending on the read depth of the sample. Some differences were observed in the performance of the tool between the two ES datasets. This can be explained by how the datasets have been generated. A caveat of the exome dataset 2 is that the FP and FN rates were based on the maximum detected number of MEIs by the tools. Therefore the accuracies and sensitivities are likely overestimated, but still reflect the comparability between tools. It should also be noted that these results were generated on samples with an average coverage of 100X (ES) and 40X (GS), and results could be different for different read depths.

Our benchmark results supported the approach of combining SCRAMble and MELT to achieve a higher MEI detection rate. This was corroborated by the results of the reanalysis of the Solve-RD and Radboudumc cohorts, where neither tool was able to detect all disease-causing MEIs. Other authors have also reported the same strategy of combining multiple tools to increase the MEIs detection rate, although not on ES data [18, 19, 35]. Methodological differences between the algorithms used to detect MEIs may explain this complementarity. To explore this possibility, we visually inspected in IGV MEI calls that differed between tools. On the one hand, MELT had the above-mentioned limitation of not detecting insertions with only a few or no DPs. On the other hand, SCRAMble was unable to detect insertions only captured by their poly-A tail, as was the case for the Alu insertion in *CC2D2A* depicted in Fig. 3c.ii, whereas MELT can leverage DPs in such cases. SCRAMble showed a slightly superior performance to MELT in the detection of clinically relevant MEIs with a clinical sensitivity of 80% and 70% in ES, respectively. Torene et al. [13] also compared SCRAMble, MELT and Mobster on clinically relevant exome MEIs and found a higher sensitivity for SCRAMble in comparison to MELT and Mobster. However, their results may be biased by the fact that only MEIs previously identified by SCRAMble were used as references.

Despite our restricted filtering strategy to focus on disease-causing MEIs, the final variant lists contained quite some low-quality calls that were discarded after inspection in IGV. In general, the reason for these calls could be inherent (1) to limitations of the short length of the reads in common sequencing technologies when mapping to complex genomic regions (e.g. regions with high homology or highly repetitive regions) or (2) to quality of some of the sample data as some samples contained a much higher number of MEI calls well above the average (Fig. 2a). The latter argument was especially true for the Solve-RD cohort, where a higher average number of calls was observed compared to Radboudumc. The Solve-RD cohort included samples sequenced in a variety of laboratories and using many different sequencing kits. This cohort is, therefore, less uniform in parameters such as read length and coverage, and is therefore likely to contain a higher number of FP calls.

Our reanalyses yielded 10 new diagnoses in previously undiagnosed patients, with an overall frequency of considering both cohorts 0.03%, which is in line with previous studies where a frequency between 0.03% and 0.04% was reported [13–15]. Although these studies differ in factors such as the selection of patient cohorts and the MEI tools used.

To the best of our knowledge, only the insertion in *BRCA2* [36, 37] and *USH2A* [13] have been previously described, while the other disease-causing events were novel findings of this study. This likely reflects the fact that MEIs are not yet routinely evaluated by clinical laboratories in patients with rare diseases and are therefore often overlooked. All patients included in our study had previously been tested by ES with negative results and therefore would not have been diagnosed without a targeted MEIs analysis. Even though ES can incorporate general structural variant (SV) callers, these are not always suitable for MEI testing [15]. In fact, two general SV callers were also applied to the Solve-RD cohort but neither Manta [38] nor InDelible [39] were able to detect these MEIs which suggests that specific MEI tools are necessary for accurate MEI detection in ES data.

Our study confirms the role of MEIs as a pathogenic mechanism in a small fraction of patients with rare diseases. The frequency reported in this study should be interpreted as the lower end of the true value. It is likely that the use of more advanced technologies, such as emerging long-read sequencing and optical genome mapping, will significantly improve the sensitivity for structural variant detection, including MEIs. Nevertheless, simultaneous detection of MEI with other variants from short-read ES data has the potential to increase diagnostic yield, reinforcing the need to incorporate MEI detection into routine diagnostic pipelines and to reanalyse exome cohorts.

### Supplementary information

Supplementary Information

Supplementary Table 1

Supplementary Table 2

Supplementary Table 3

Supplementary Table 4

Supplementary Table 5

Supplementary Table 6

Supplementary Table 7

Supplementary Table 8

Supplementary Table 9

Supplementary Table 10

Solve-RD consortium

## Data Availability

All data generated and/or analysed during this study are available through any reasonable request from the corresponding author.
