# Correction: Mobile element insertions in rare diseases: a comparative benchmark and reanalysis of 60,000 exome samples

**DOI:** 10.1038/s41431-023-01492-9

**Published:** 2023-11-16

**Authors:** Robin Wijngaard, German Demidov, Luke O’Gorman, Jordi Corominas-Galbany, Burcu Yaldiz, Wouter Steyaert, Elke de Boer, Lisenka E. L. M. Vissers, Erik-Jan Kamsteeg, Rolph Pfundt, Hilde Swinkels, Amber den Ouden, Iris B. A. W. te Paske, Richarda M. de Voer, Laurence Faivre, Anne-Sophie Denommé-Pichon, Yannis Duffourd, Antonio Vitobello, Martin Chevarin, Volker Straub, Ana Töpf, Anneke J. van der Kooi, Francesca Magrinelli, Clarissa Rocca, Michael G. Hanna, Jana Vandrovcova, Solve-RD consortium, Stephan Ossowski, Steven Laurie, Christian Gilissen

**Affiliations:** 1 Department of Human Genetics, Radboud University Medical Center, Nijmegen, The Netherlands; 2 Donders Institute for Brain, Cognition and Behaviour, Radboud University, Nijmegen, The Netherlands; 3 Universitätsklinikum Tübingen – Institut für Medizinische Genetik und angewandte Genomik, Tübingen, Germany; 4 Radboud Institute for Molecular Life Sciences, Radboud University Medical Center, Nijmegen, The Netherlands; 5 Centre de Référence Maladies Rares “Anomalies du développement et syndromes malformatifs”, Centre de Génétique, FHU-TRANSLAD et Institut GIMI, CHU Dijon Bourgogne, Dijon, France; 6 UMR1231-Inserm, Génétique des Anomalies du développement, Université de Bourgogne Franche-Comté, Dijon, France; 7 Laboratoire de Génétique chromosomique et moléculaire, UF6254 Innovation en diagnostic génomique des maladies rares, Centre Hospitalier Universitaire de Dijon, Dijon, France; 8 John Walton Muscular Dystrophy Research Centre, Translational and Clinical Research Institute, Newcastle University and Newcastle Hospitals NHS Foundation Trust, Newcastle upon Tyne, UK; 9 Department of Neurology, Amsterdam UMC, University of Amsterdam, Amsterdam Neuroscience, Amsterdam, The Netherlands; 10 Department of Clinical and Movement Neurosciences, UCL Queen Square Institute of Neurology, London, UK; 11 Department of Neuromuscular Diseases, UCL Queen Square Institute of Neurology, London, UK; 12 Clinical Pharmacology, William Harvey Research Institute, School of Medicine and Dentistry, Queen Mary University of London, London, UK; 13 Centro Nacional de Análisis Genómico (CNAG), Centre for Genomic Regulation (CRG), Barcelona Institute of Science and Technology (BIST), Barcelona, Spain

**Keywords:** Genetics, Computational biology and bioinformatics

Correction to: *European Journal of Human Genetics* 10.1038/s41431-023-01478-7, published online 19 October 2023

In this article the full list of members of the consortium was missing. It has now been uploaded as supplementary information.

The original article has been corrected.

### Supplementary information

Supplementary Information

