# Supplementary material for: Mobile element insertions in rare diseases: a comparative benchmark and reanalysis of 60,000 exome samples
Source: Eur J Hum Genet. 2023 Oct 19;32(2):200–8. doi: 10.1038/s41431-023-01478-7 (PMC10853235; doi:10.1038/s41431-023-01478-7)
Supplement: Supplementary file 1 — Supplementary Information [file 41431_2023_1478_MOESM1_ESM.docx]

Supplementary Information

Mobile element insertions in rare diseases: a comparative benchmark and reanalysis of 60,000 exome samples

Robin Wijngaard, German Demidov, Luke O’Gorman, Jordi Corominas-Galbany, Burcu Yaldiz, Wouter Steyaert, Elke de Boer, Lisenka E. L. M. Vissers, Erik-Jan Kamsteeg, Rolph Pfundt, Hilde Swinkels, Amber den Ouden, Iris B. A. W. te Paske, Richarda M. de Voer, Laurence Faivre, Anne-Sophie Denommé-Pichon, Yannis Duffourd, Antonio Vitobello, Martin Chevarin, Volker Straub, Ana Töpf, Anneke J. van der Kooi, Francesca Magrinelli, Clarissa Rocca, Michael G. Hanna, Jana Vandrovcova, Solve-RD consortium, Stephan Ossowski, Steven Laurie, Christian Gilissen.

Table of Contents

[Supplementary Materials and Methods 3](#_Toc143885250)

[Exome benchmark datasets creation 3](#_Toc143885251)

[Exome dataset 1 3](#_Toc143885252)

[Exome dataset 2 4](#_Toc143885253)

[MEI validation 5](#_Toc143885254)

[*NIPBL* Alu insertion 5](#_Toc143885255)

[*COL6A2* Alu insertion 5](#_Toc143885256)

[*NKX2-1* Alu insertion 6](#_Toc143885257)

[Radboudumc MEIs 6](#_Toc143885258)

[Supplementary Results 7](#_Toc143885259)

[Detailed description of the three MEI cases depicted in Figure 3 7](#_Toc143885260)

[Case 1 (Solve-RD): *De novo* Alu insertion in *NIPBL* gene 7](#_Toc143885261)

[Case 2 (Solve-RD): Inherited Alu insertion in *COL6A2* gene 7](#_Toc143885262)

[Case 3 (Radboudumc): Inherited Alu insertion in *CC2D2A* gene 8](#_Toc143885263)

[Supplementary Figures 9](#_Toc143885264)

[Supplementary Figure 1 9](#_Toc143885265)

[Supplementary Figure 2 10](#_Toc143885266)

[Supplementary Figure 3. 11](#_Toc143885267)

[References 13](#_Toc143885268)

# Supplementary Materials and Methods

## Exome benchmark datasets creation

### Exome dataset 1

To validate accuracy of the mobile element insertion (MEI) detection tools, a benchmark set of 20 exome samples was employed**.** Two samples, NA19240 and HG00733, part of the Phase 3 release from the 1,000 Genomes project, were retrieved through The International Genome Sample Resource (IGSR) Collection (1, 2). Data from an additional two samples (HG002 and HG005) were obtained from the Genome in a Bottle Consortium (3). The remaining 16 samples were sequenced at the Radboudumc (4). All samples had a read length ranging from 76 to 150 nucleotides and were aligned to the GRCh37 assembly. The average coverage across the samples was approximately 92X. Exon boundaries were established by overlapping the target file from the Phase 3 of the 1000 Genomes project and the AgilentV5 target file.

For the same set of samples, long-read sequencing data using PacBio HiFi reads were available from the year 1 release of the Human Pangenome Reference Consortium (5) (https://github.com/human-pangenomics/HPP_Year1_Assemblies) or were sequenced at the Radboudumc. Samples had a read length of approximately 15,000 nucleotides and an average coverage of around 31X.

To curate MEIs within these samples, we employed PALMER with its default parameters (6). The resulting output was subjected to filtering using the recommended cut-offs of ≥ 1 high-confident supporting reads and ≥ 3 supporting reads. This dataset encompassed a total of 46,183 MEIs spanning the entire genome. Genomic positions were converted from GRCh38 to GRCh37 by utilizing the University of California Santa Cruz (UCSC) LiftOver tool. Conversion failed on 4,437 calls, which were subsequently excluded from further analysis. We then filtered on MEIs falling within target regions, defined as those situated within exons or within a 50 bp window around exome targets. This resulted in a final set of 256 MEIs, used as reference set for benchmarking, with an average of 12.8 per sample. An overview of the number of calls per sample and categorised by MEI type is shown in **Supplementary Table 10**.

### Exome dataset 2

In parallel we generated a high-confidence MEI call set using data from 100 exome parent-child trios sequenced at the Radboudumc. Samples had a read length of between 100 and 150 nucleotides, with an average coverage of 108X. Exome capture was performed using Agilent SureSelect v4 (n = 39) or Agilent SureSelect v5 (n = 61).

All tools included in our study were run with default settings on these samples, and calls were merged into one dataset. To avoid the same MEI being considered as two independent calls, the tool call sets were merged assuming an imprecision of +/- 100 bp from the predicted insertion breakpoint. The complete MEI dataset included 12,758 different MEI calls. We then filtered to MEIs falling within the target regions, leaving 5,599 calls. MEI inheritance was characterised by considering a MEI as inherited if the same type of mobile element (Alu, L1, SVA) overlapped between the proband and parent data by +/- 50 bp.

In order to obtain a high-confidence reference dataset, this complete MEI dataset was filtered to exclude potential false positive (FP) calls. We assumed that almost all MEIs in the dataset should be inherited, given the low frequency of *de novo* events. However, a significant number of *de novo* calls was observed due to low coverage or low-quality parent data. Therefore, we only excluded MEI sites that were found as *de novo* in all samples where they were present*.* We also excluded calls that were found in more than five samples only by a single method, as it might reflect an inherent issue with the method. The final calls were manually curated by visual inspection in the Integrative Genomics Viewer (IGV). MEIs detected in low-quality mapping regions were also excluded. The filtered dataset (high-confidence call set) contained 1,111 calls and was used for tool comparison (**Supplementary Table 10**).

To validate our filtering approach, we tested the distribution of the number of MEI calls per sample. The number of MEI calls per sample followed a normal distribution (Shapiro-wilk p>0.05), with an average of 11 calls per sample. In addition, we checked for MEIs included in the retrotransposon insertion polymorphisms in humans (dbRIP) database (7), *i.e.* known human polymorphisms. 636 MEI calls in the unfiltered dataset of 5,599 calls were described in dbRIP, of which 631 (99.2%) were retained in the final dataset of 1,111 calls, demonstrating that our approach was able to keep true positive calls in the dataset.

## MEI validation

### *NIPBL* Alu insertion

Validation was performed at the Laboratoire de Génétique chromosomique et moléculaire, UF6254 Innovation en diagnostic génomique des maladies rares, Centre Hospitalier Universitaire de Dijon, France. Exons 26 and 27 of *NIPBL* were amplified using a standard PCR protocol. PCR products were checked by gel electrophoresis which revealed three bands for the index case and only one band for his parents, confirming that the MEI is *de novo.* Each band of the index case's PCR product was excised from agarose gel, re-amplified and Sanger sequenced using the same primers.

### *COL6A2* Alu insertion

Validation was performed at the John Walton Muscular Dystrophy Research Centre, Newcastle upon Tyne, UK. Allele specific primers were designed around the exon-intron junction of ex-in10 of *COL6A2* where the MEI was expected, so that they would amplify exclusively the allele carrying the Alu insertion. PCR products were checked by gel electrophoresis and revealed a band only in the proband and affected family members but not in the control DNA, or unaffected individuals from the same family.

### *NKX2-1* Alu insertion

The MEI identified in *NKX2-1* was validated at UCL Queen Square Institute of Neurology, University College London, London, United Kingdom. Long-distance primers were designed with Primer-BLAST to amplify bidirectionally the *NKX2-1* region carrying the identified Alu element insertion (Fw: 5’-CACTGCGTCTTTTGGTTCGA-3’, Rv: 5’-GTTTGCCGTCTTTCACCAGG-3’; amplicon size of the wild-type allele: 706bp). PCR was performed with optimized conditions. PCR products were loaded into 2% agarose gel and underwent electrophoresis. The gel band of approximately 1000 bp predicted to carry the allele with the Alu element insertion was extracted from gel. DNA purified from the latter was cloned in chemically competent *E. coli*, and the recombinant plasmid underwent Sanger sequencing with the above-mentioned primers. The proband’s parents tested negative for the Alu insertion, confirming that it was a *de novo* event.

### Radboudumc MEIs

All Radboudumc results were validated inhouse. Primers were designed to amplify DNA fragments between 4 and 13 kb in length around the predicted MEI insertion site. Amplicons were amplified by long-range PCR. The products were checked with a gDNA tapestation. When correct amplification was observed, a PacBio library was prepared and the library was sequenced on a PacBio Sequel I. The reads obtained were CCS3 mapped and data were reviewed in IGV.

# Supplementary Results

## Detailed description of the three MEI cases depicted in **Figure 3**

### Case 1 (Solve-RD): *De novo* Alu insertion in *NIPBL* gene

A 9-year-old proband, with no relevant family history, who had been referred at 29 months old to the genetics department presenting with global developmental delay, growth retardation, microcephaly, axial hypotonia, bilateral ptosis, prominent symphysis, bilateral single transverse palmar crease, clinodactyly of the fifth finger and hypoplasia of the thumbs. Cardiac ultrasound and electrocardiogram evidenced an aortic arch with crenellated appearance without any criteria of coarctation. Cerebral magnetic resonance imaging (MRI) showed a thin but complete corpus callosum and an unusual signal of the anterior pituitary gland related to unusual fluid formation repressing the anterior pituitary gland. Radiography of the hands showed a hypoplasia of the first metacarpal. These findings established the clinical diagnosis of Cornelia de Lange syndrome. At that moment, exome sequencing was performed with negative results. The MEI analysis undertaken here revealed an intronic Alu insertion in the *NIPBL* gene, associated with Cornelia de Lange syndrome I. The absence of the insertion in the parents of the child confirmed that the insertion was *de novo* in the patient (**Figure 3a**).

### Case 2 (Solve-RD): Inherited Alu insertion in *COL6A2* gene

A 59-year-old male who had childhood onset of a characteristic Bethlem myopathy phenotype presenting with proximal weakness and contractures including the flexors of the fingers and elbows. Electromyography displayed myopathic abnormalities and muscle MRI pattern was typical of a collagen VI-related disorders. There were four additional affected relatives over two generations in the family, demonstrating an autosomal dominant pattern of inheritance. After negative ES, our MEI analysis revealed an Alu insertion in the *COL6A2* gene. The variant co-segregated correctly with the disease phenotype in the family (**Figure 3b.i**).

### Case 3 (Radboudumc): Inherited Alu insertion in *CC2D2A* gene

Two sisters with a similar neurological disorder, characterised by development problems, mild motor (cerebellar) problems, ataxia, eye movement disorders and a so called “molar tooth sign” in MRI. In our MEI analysis, an Alu insertion in the *CC2D2A* gene was initially only found in one of the sisters. However, a poly-A tail was also visible in the other sister in IGV suggesting a possible MEI (**Figure 3c.ii**). Confirmatory PCR analysis indicated the presence of the MEI in both sisters, who also had a missense single nucleotide variant (SNV) (NM_001080522.2(CC2D2A):c.4465G>C) classified as variant of unknown significance. The variants were confirmed to be in compound heterozygous phase with the MEI being inherited from the father, and the SNV from the mother. These findings supported the diagnosis of Joubert syndrome 9, an autosomal recessive disorder that matched the phenotype of the patients and the suspected pattern of inheritance.

# Supplementary Figures


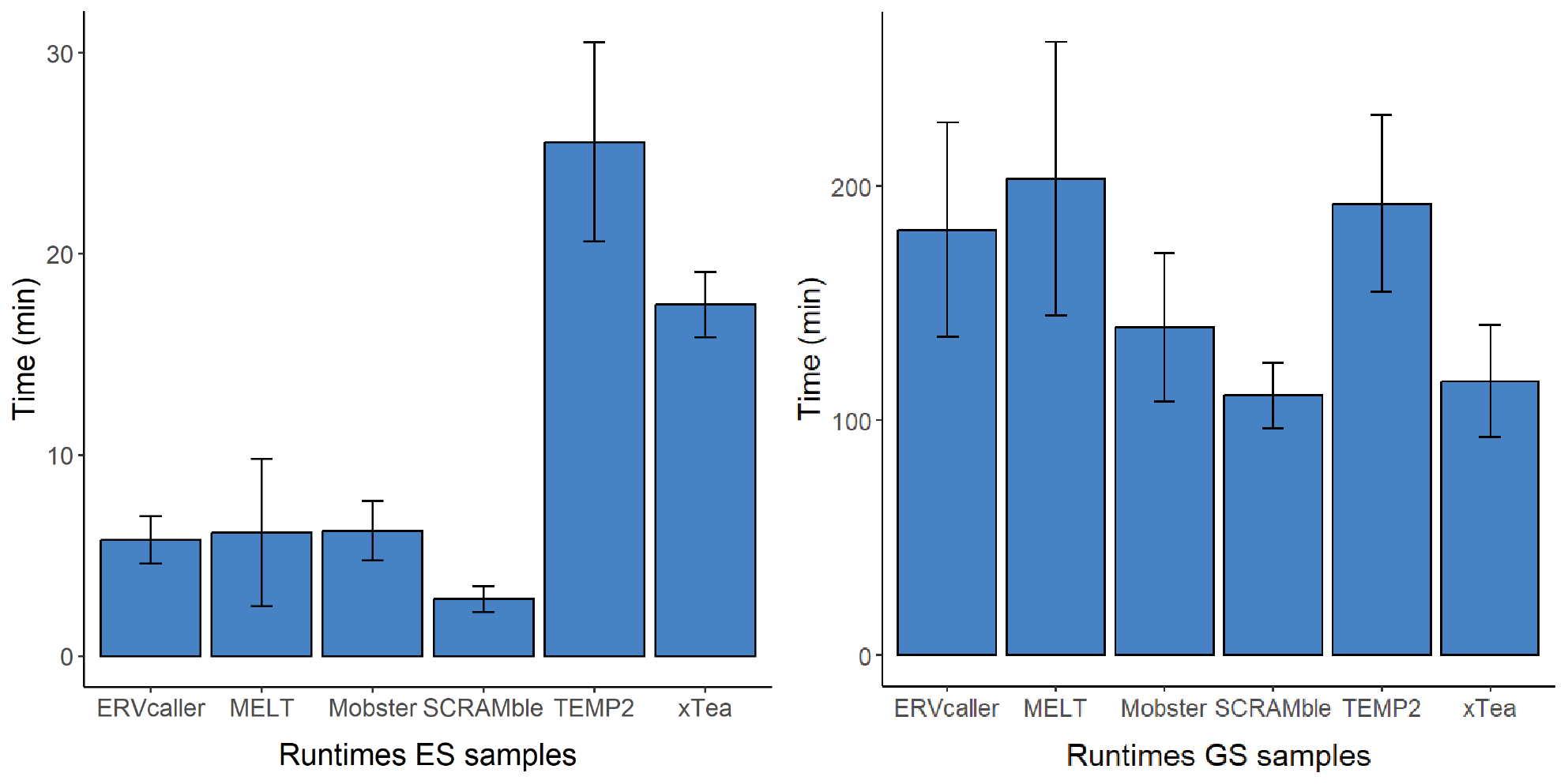


Supplementary Figure 1**.** Runtimes on exome sequencing data (n=300) and genome sequencing data (n=2). Tool were run with 1 CPU (MELT, Mobster, SCRAMble, TEMP2) or 16 CPU (ERVcaller and xTea). In the case of xTea, the time represents the sum of the time for each mobile element, as they are executed independently.


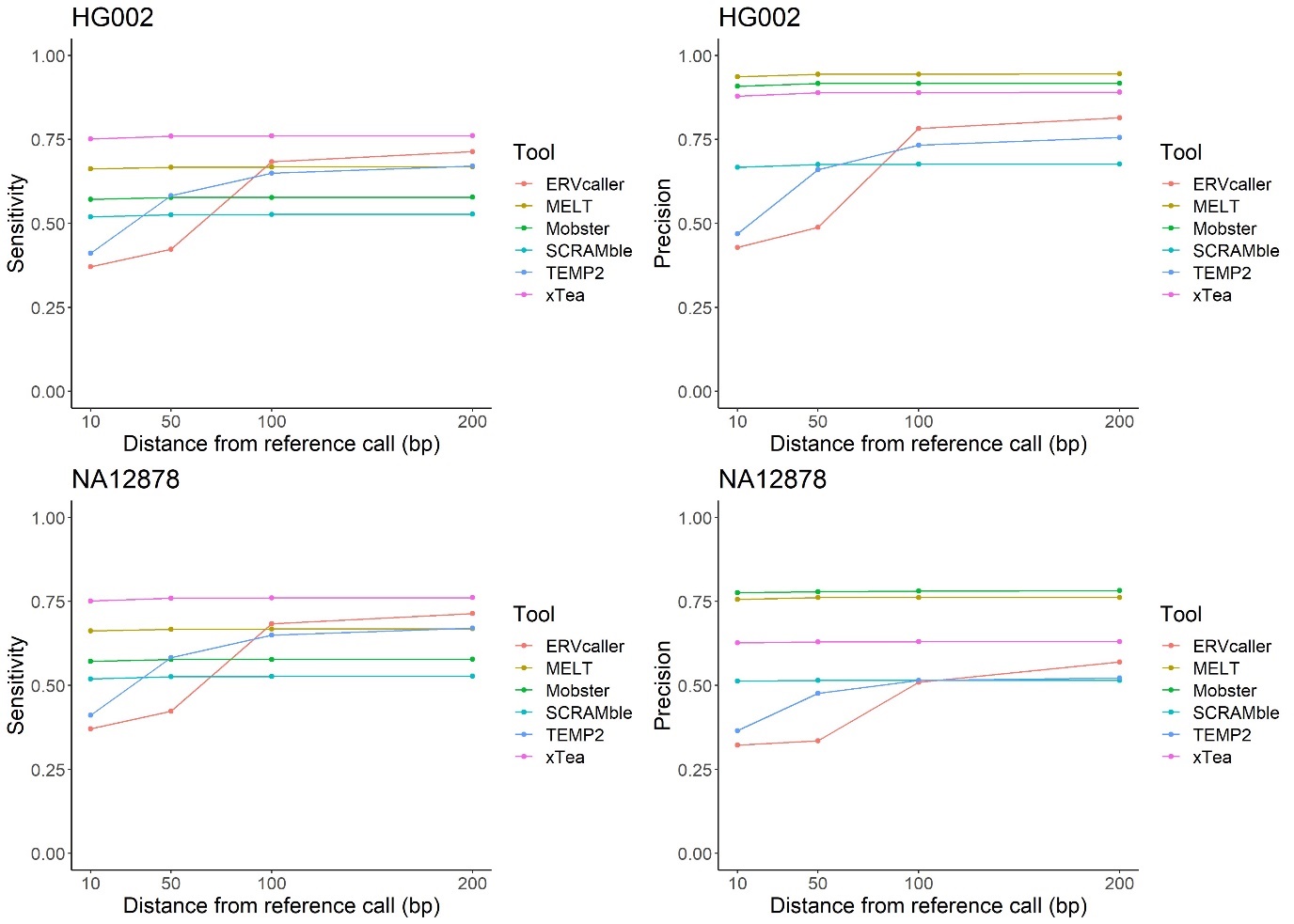


Supplementary Figure 2. Sensitivities and precisions obtained when considering correct mobile element insertion (MEI) calls within various window sizes (10, 50, 100 or 200 bp) around the target site duplication (TSD) of a reference insertion site. Both HG002 and NA12878 genome samples are represented. MELT, Mobster, SCRAMble and xTea demonstrated high performance in predicting MEIs within a 10 bp window of the reference call. In contrast, ERVcaller and TEMP2 exhibited significantly improved sensitivity and precision as the distance from the reference call increased, indicating less accurate prediction of MEI breakpoints by these tools.


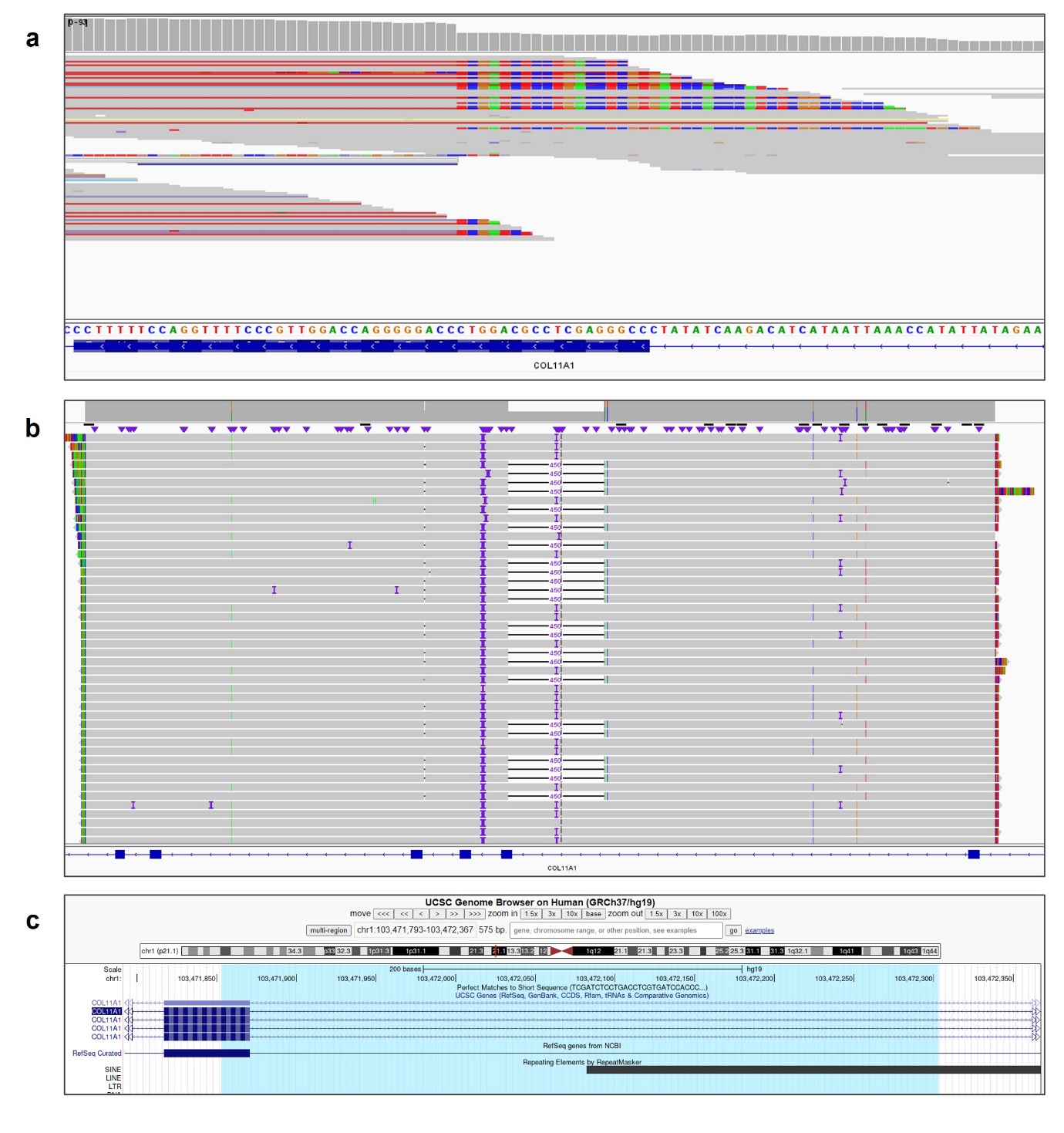


Supplementary Figure 3. Detected deletion in the *COL11A1* gene in our mobile element insertion (MEI) analysis. **a** Screen capture from the integrative genomics viewer (IGV) showing the deletion as observed in the ES data. Both split reads (SRs) and discordant pairs (DP) are present. The red colour of the DP denotes a larger than expected inferred insertion size, evidencing a possible deletion. **b** Confirmation results by amplicon long-read sequencing, showing a heterozygous deletion of 450 bp. **c** Screen capture of the deleted region (highlighted in light blue) in the University of California at Santa Cruz (UCSC) Genome Browser. Note that the end of the deletion occurs within a repeat region of the reference genome, specifically within an ancient Alu insertion. This leads to sequence reads containing both a segment of the exon sequence coupled with a fragment of a mobile element sequence. When aligned to the reference genome, reads aligning to the exon will manifest soft clipped bases containing Alu element sequence.

# References

1. Genomes Project C, Auton A, Brooks LD, Durbin RM, Garrison EP, Kang HM, et al. A global reference for human genetic variation. Nature. 2015;526(7571):68-74.

2. Fairley S, Lowy-Gallego E, Perry E, Flicek P. The International Genome Sample Resource (IGSR) collection of open human genomic variation resources. Nucleic Acids Res. 2020;48(D1):D941-D7.

3. Zook JM, Catoe D, McDaniel J, Vang L, Spies N, Sidow A, et al. Extensive sequencing of seven human genomes to characterize benchmark reference materials. Sci Data. 2016;3:160025.

4. Kucuk E, van der Sanden B, O'Gorman L, Kwint M, Derks R, Wenger AM, et al. Comprehensive de novo mutation discovery with HiFi long-read sequencing. Genome Med. 2023;15(1):34.

5. Liao WW, Asri M, Ebler J, Doerr D, Haukness M, Hickey G, et al. A draft human pangenome reference. Nature. 2023;617(7960):312-24.

6. McDonald TL, Zhou W, Castro CP, Mumm C, Switzenberg JA, Mills RE, et al. Cas9 targeted enrichment of mobile elements using nanopore sequencing. Nat Commun. 2021;12(1):3586.

7. Wang J, Song L, Grover D, Azrak S, Batzer MA, Liang P. dbRIP: a highly integrated database of retrotransposon insertion polymorphisms in humans. Hum Mutat. 2006;27(4):323-9.
